# Effectiveness of online counselling during COVID-19 in Zambia: clients' and therapists' perspectives

**DOI:** 10.1186/s40359-024-01614-y

**Published:** 2024-03-08

**Authors:** Choongo Mulungu, Tafadzwa Mindu, Kelvin Mulungu

**Affiliations:** 1 University of Lusaka/ Ministry of Health – Lusaka District Health Office, Lusaka, Zambia; 2 International Maize and Wheat Improvement Center (CIMMYT), Lusaka, Zambia; 3 School of Nursing and Public Health, Univeristy of KwaZulu-Natal, Durban, South Africa

**Keywords:** Online Counselling, Applications, Subjective well-being, Clients, Therapists

## Abstract

Population-based studies have shown that a growing number of counsellors have been providing counselling via the Internet. There are mixed findings regarding the effectiveness of online counselling compared to traditional face-to-face counselling and other modalities. During the COVID-19 outbreak, online counselling services were advanced as the only safe means of attending to mental health conditions, especially during the lockdowns. However, the effectiveness of online counselling remains unclear, especially in developing countries with low digital literacy and poor Internet connectivity. This study's main purpose was to investigate and determine the clients' and therapists' perspectives on the effectiveness of online counselling during the pandemic based on the level of preparedness, mode of delivery, and challenges faced. The study used mixed methods employing a parallel convergent design and collected data from 284 participants (44 therapists and 240 counselling clients). The study found that therapists were not prepared to offer online counselling services, but were compelled by high demands for counselling services from clients during the pandemic. Both clients and therapists cited the cost of data bundles to connect to the Internet as a major barrier. Furthermore, both clients and therapists indicated that the most effective platform was video conferencing. The findings also show that therapists observed slowed progress in the client's recovery due to clients missing sessions. Logistic regression results on factors associated with preparedness and positive perception of online counselling showed that older therapists and lay counsellors were less likely to be prepared and evaluate sessions as less effective, respectively. Living in Lusaka (the capital) was associated with higher odds of perceiving online counselling as effective. Client results showed that older clients had lower odds of being prepared, and the counsellor being prepared increased the odds of the online counselling being perceived as effective. The researchers conclude that online counselling, in the context of a developing country, is ineffective because of the cost of Internet connection, the multiplicity of information communication technology (ICT) channels, low digital literacy, and lack of knowledge or experience using these platforms.

**Supplementary Information:**

The online version contains supplementary material available at 10.1186/s40359-024-01614-y.

## Background

In the last 20–25 years, the world has seen electronic mental health services (e-mental) emerging as a promising avenue for intervention because of their advantages in accessibility and efficiency over traditional service provision [1, 2]. In particular, these services have been advocated for use in public health strategies for young people aged 10 to 24 years, who have the highest risk for developing emotional and mental health problems [3] and are more likely to delay seeking professional treatment [4] because of a number of help-seeking obstacles such as stigmatization, undermining the problem, self-dependence, in-accessible health services, etc. [5].

Cyberpsychology is a field of psychology that involves studying human experiences (cognitive, emotional, and behavioral) related to or affected by developing technologies [6]. One of the areas of cyberpsychology is online counselling, also referred to as e-therapy, e-counseling, e-mental, or cyber therapy. Richards and Vigan'o defined online counselling as a mental health intervention between the counselee/client and counsellor or therapist using digital technologies such as computers or smartphones [7]. Mallen and Vogel provide a comprehensive definition of online counselling: "any delivery of mental and behavioral health services, including but not limited to therapy, consultation, and psych education, by a licensed practitioner to a client in a non-face-to-face setting through distance communication technologies such as the telephone, asynchronous e-mail, synchronous chat, and video conferencing" [8]. In online counselling, there is a therapeutic relationship between the counselee and the counsellor, who are in different locations but communicate via the Internet or computer technologies [9, 10].

Many researchers have debated the nature of online counselling [11–14]. The advent of the COVID-19 pandemic demanded a change in many practices, including counselling, which saw therapists adopting online counselling. Many countries imposed regulations for working from home, studying from home, social distancing, physical distancing, etc. Furthermore, these adjustments had the potential to trigger mental health symptoms such as anxiety, depression, and stress in some people [15, 16]. Zambia was not exempted from these changes, as it introduced restrictions that demanded patients/clients generally considered to have non-emergency conditions, such as mental health, diabetes and hypertension to stay at home (MOH, Zambia 2020). These restrictions led to the uptake of online counselling. As Internet-based interventions continue to increase in number, scope, and usage, there is a need for research to determine how these interventions can be applied to achieve the best possible outcomes during pandemics in developing countries such as Zambia. Specifically, it is important to understand the perspectives of both therapists and clients regarding the effectiveness of online counselling in a developing-country context.

There are mixed findings regarding the effectiveness of online counselling when compared to traditional face-to-face counselling and other modalities. For example, a narrative and critical review of the literature on online counselling found that a growing body of knowledge to date is almost unanimous in showing that online counselling can have a similar impact and is capable of replicating the facilitative conditions as face-to-face encounters [7]. Similarly, other studies have shown that online counselling can be as effective as face-to-face counselling [9, 17]. Online counselling is a convenient service because it can be provided at any time of the day, and clients can send messages whenever they feel most in need of or interested in therapy [18]. Essentially, online counselling is accessible from any corner of the world as long as Internet access is available [19].

The following studies [9, 17, 18] reviewed the use of emails and chat based platforms, each study viewed these methods as being advantageous or effective just as face to face therapy. More specifically [18] found emails and chat platforms to be effective as methods for complementing video therapy or in person therapy, they state that emails serve a good purpose for those days in between sessions when the therapists make follow up, or when the client has just finished therapy and is now recovered, the therapist will then make follow ups via email or texts, easing the follow-up process. The other studies (systematic reviews) by [9, 17] report that emails are as effective as face to face in various contexts of therapy. They discuss findings from studies on smoke cessation, eating disorders, anxiety etc. In all their examples the findings show no significant differences in effectiveness between emails and face to face methods. The novelty on online communication removes certain barriers such as being shy, anxious, or busy schedules that prevent clients from showing up.

Still, other studies report the inability of emails and texts to offer human interaction as a downside of online counselling [19]. Barak et al.’s review has other studies that report negatively on emails. They state that emails or text lack physical presence in human communication and may decrease the sense of intimacy, trust, and commitment in the therapeutic relationship [17]. Consequently, this may weaken the development of a therapeutic foundation between the counselor and the client. The absence of visual and vocal cues, such as facial expressions, body language, and voice tone, can result in greater potential for miscommunication during text-based session. These crucial emotions (sighs, frustrated emotions, terseness, and irritation) are not sharable during texts or emails [20].

A systematic review by Backhaus et al. discussed the feasibility of video-conferencing practices in online counseling [21]. They found that these are feasible given their ability to link participants and the therapist in real time. While this is valuable information, the only downside is that most studies reporting on online counselling are conducted in developed countries [10, 22–30] [8, 17–25], where there is good infrastructure to support high quality video conferencing and high digital literacy. This creates a dearth of literature on the African context. The growth of the Internet in Africa and Zambia in particular, has created excellent opportunities for providing accessible counselling via computers and phones. Many counsellors have begun to grasp the enormous opportunity to reach multiple groups of underserved populations who will benefit from Internet/Web-based services. ZICTA [31] indicates that Zambia has 10.4 million people with Internet access, representing about 58% penetration rate. Most of these households are in Lusaka province which has 18% of the population, followed by Copperbelt province with 15%.

The COVID-19 pandemic in Zambia showed that many Zambians had turned to online platforms to seek counselling services, and at the time, numerous online platforms, such as Facebook pages, were observed to provide such services. While online platforms offer different types of services to their clients in Zambia what is not known is how effective online counselling services are to the therapists and their clients.

Given that many therapists and patients resorted to the use of online platforms to access their clients during the pandemic, this study sought to establish how effective these online sessions were. Hence, the main purpose of this study was to examine and validate the opinions of clients and therapists regarding the effectiveness of online counseling during the COVID-19 pandemic in Zambia. The sub objectives for the study were to establish the therapists' preparedness to offer online counselling sessions during the pandemic, to explore the limitations faced by clients and therapists during online counselling, to establish the applications explored for the provision of online counselling during the pandemic, and to establish the factors influencing preparedness for and perceived effectiveness of online counselling.

## Methods

The study used mixed methods employing a parallel convergent design to conduct this research with the support of evaluative qualitative data collection techniques. It involved non-experimental procedures for the two groups in the study, namely, the online therapist and client groups. Data were collected in two phases: a questionnaire and a focus group discussion. We chose to use mixed methodology so that we could get an analytical description of the problem as well a deeper understanding of the factors affecting the use of online therapy. The mixed methodology allowed us to gather statistics and then draw deeper conclusions on why certain aspects of online counselling were preferred by each participant. Our objectives allowed us to probe the questions quantitatively and qualitatively. Some specific objectives such as establishing the preparedness of therapist needed more than just a yes or no answer, we also wanted to establish the reasons why they were not prepared. Yes and no answers were good in that they gave us statistics on how many participants were prepared, but the qualitative aspect enabled us to understand the why and how aspect. This is similar to the objective on effectiveness, which also needed us to draw an understanding of how effectiveness was determined by the participants. Hence the mixed method analysis allowed us to draw a deeper understanding of online counselling effectiveness.

### Participants

The study participants were purposively selected from counselling service providers recognized by the Psychology Association of Zambia (PAZ) drawn from across Zambia. We focused on the clients and counsellors in Zambia. This association was chosen because it has a compiled mental health service provider directory, which made it easy for the researcher to randomly sample from this list and extend the questions to their clients. The questions were attended through the Internet during the COVID-19 outbreak. The researcher emailed the counsellors and followed up using social media (WhatsApp) to contact their clients, whose identity was withheld. In addition, the researchers attended several online group counselling sessions organized by service providers to make announcements about the research. Simple random sampling was applied to select counsellors from the PAZ directory. Sixty-two therapists were selected; however, due to dropouts and non-responsiveness, only 44 participated in the study. Informed written consent was obtained from the therapists and clients to participate in the study. In total, 244 participants (240 clients and 44 counsellors) consented to participate in this study. Most of the clients were female (*n* = 180, *p* = 75%) and most of the therapists were also female (*n* = 27, *p* = 61%). The therapists were from different types of practices: psychosocial counsellors (*n* = 10), psychiatrists (*n* = 6), psychologists (*n* = 8), followed by religious leaders (*n* = 5), social worker (*n* = 7), lay counsellor (*n* = 3) and mental health nurse (*n* = 2). In terms of work setting, most therapists worked at health or mental clinics (*n* = 14), hospitals (*n* = 8), university (*n* = 7), individual practice (*n* = 2), group practice (*n* = 2) and military (*n* = 2).

### Procedure

The participants were informed about the study procedure, such as interventions, data collection, and their right to withdraw. The participants were asked to read and sign an online informed consent form before proceeding with the questionnaires. After receiving informed consent forms, the questionnaires were sent to them by therapists identified through the Psychology Association of Zambia (PAZ) Directory. They were then allocated to one of two groups (one for therapists and the other for clients connected to therapists). Upon completing the questionnaire, the clients were put into groups to discuss their experiences and perceived effectiveness of this relatively new approach to service delivery in the country.

Since the first case was confirmed in Zambia on March 18, 2020, the country experienced four waves of COVID-19: an initial wave beginning in July 2020, a much larger second wave during January and February 2021, an even larger third wave during June and July 2021, and a fourth wave during December 2021 and January 2022. The second wave was most likely fueled by the South African variant of the virus (B.1.351, beta), which was first observed in Zambia in December 2020. As of June 24, 2022, there was more than 324,922 confirmed cases and 4,000 deaths from COVID-19 in the country. The study took interest to assess the effectiveness of online counseling after the counseling having been provided starting in the second wave especial necessitated by restrictions imposed by the government in order to manage the scarce in cases.

This study was done after the fourth wave and concentrated on mental health services offered and accessed online to determine experiences and in turn assess efficacy. Data was collected between May 2022 and September 2022. The data collection took longer than expected especially considering the fact that we used an online form for quantitative data and the focus group discussions. Quantitative data was collected first and this took the longest as it was dependent on individual convenience. Within the stated period, focus group discussions were done. In the Supplementary files, we have included a questionnaire (Supplementary 1) and a Focus Group Discussion Guide (Supplementary 2) just to highlight what kind of data we collected.

### Study participants

All therapists were registered with the Psychology Association of Zambia for the services they offered. This meant that by the time of data collection, these therapists had been offering services for more than two years (based on the year of publication of the PAZ Directory). Before the study, therapists were engaged to establish the services they offered online and their modes of delivery to select only those that provided online services of interest to the study. Furthermore, the selected therapists were requested to avail the clients they attended during the pandemic. The clients were required to meet the inclusion criteria of age and consent and had attended counselling sessions from a listed therapy provider during the period under review. This was similar to snowball sampling. Counsellors helped send the link to their clients as a quality assessment for the services they provided.

These clients responded to the online questionnaire, and later a follow-up was made on the same clients through the counsellors to invite them to an information session about a focus group discussion to probe further online counselling. The nature of the discussion was explained via a zoom call meeting, in which it was made clear that the client’s identity was not required; therefore, participants were advised to join with a pseudonym and that there was no need for participants to use their camera during the group discussion. Further, it was explained that the call would be recorded, but the researcher would only retain a writeup transcript that would be used for the originality of thoughts. Clients were asked to save a zoom link shared in the chat during the virtual meeting (information meeting via zoom). Seven groups of eight clients participated in recurring meeting schedules one after the other. However, one group was not considered, as it had only four participants (the researcher and three clients). This study only presented submissions from six groups that had eight clients with the researchers.

### Research instruments

The instruments used in this study were a questionnaire for quantitative data collection and a focus group discussion (FGDs) guide for qualitative data collection. The researcher primarily collected data through online questionnaires to obtain detailed information. The questionnaires were used by both clients and therapists because of their ability to collect data from a large sample and their rigidity against biases. Questionnaires also served time during data collection and analysis as the data were collected from different samples. (See Supplementary file 1 for the questionnaire used in this study).

A focus group discussion guide was used in this study (See Supplementary file 2). The use of FGDs to generate data in qualitative research was advantageous because it gave more latitude to respondents and interviewers, allowing them to explore issues emerging from the research. Discussions were built around the emerging responses of each client rather than being bound by predetermined issues. A structure of questions was made through a focus group discussion guide, but the build-up to the discussion was through the researcher asking for clarity and probing further on emerging issues.

The group discussions lasted 40 min (the researcher used the free version of zoom), but it must be noted that they were delays in starting due to poor time keeping by clients. The meetings only started after a minimum of six clients had joined the virtual meeting. Others that joined while the meeting was on were accepted, but only a maximum of two, while others were requested to join the next meeting. The discussion was guided by the focus group discussion guide, which asked open-ended questions, and the researcher received opinions from 3–4 to people while asking follow-up questions as need rose. Debates were allowed and a consensus was established on key issues.

### Data analysis

For the qualitative data, the thematic analysis approach described by Braun and Clarke (2006) was used to understand participants’ perceptions of the characteristics of online counselling and factors moderating its efficacy. Thematic analysis involved developing familiarity with data, generating initial codes, searching for themes, reviewing themes, defining and naming themes, and producing a report of findings.

The researcher read all transcripts, and then consolidated two focus group discussion transcripts (i.e., for clients and the other for therapists). The researcher manually defined a set of preliminary codes, which did not necessarily conform to the language used in the interview guide. Qualitative themes were rated only once for each participant describing them, irrespective of the number of times that the theme was identified across the discussion groups. The final stages of the analysis involved reviewing themes and subthemes to ensure that the coded extracts were valid, logical, and reasoned.

A frequency count was applied to specific variables to identify their commonality in providing a consolidated list of experiences and suggestions, wherein the researcher sought to establish those with higher occurrence. Ratings of very strong, strong, moderate, and weak were given if > 80%, > 60%, > 50%, and > 30% of the participants endorsed the theme, respectively. Given the small sample size of this study, themes endorsed by fewer than two participants were excluded from the analysis due to low generality. The ratings were used in the study as a way of summarizing the many responses into themes for analysis. We adopted the ratings from previous studies. For example, Navarro et al. [32, 33] used the same ratings in their analysis.

Quantitative data collected from the online questionnaire were analyzed using descriptive statistics in the form of percentages and frequencies. Statistical Package for the Social Sciences (SPSS) version 23 was used to enhance the analysis. Computer-generated tables of frequencies and percentages were used to describe the variables that were presented in the form of tables and figures. This allowed for the objective interpretation of valid generalizations, conclusions, and recommendations for future studies.

Finally, to understand the factors associated with preparedness and positive perception (i.e., that it is efficacious) for counsellors and clients, a logistic regression model was estimated. In the first model for therapists, preparedness was categorized as one if the therapist was prepared and zero if they were not or somewhat prepared. In the second model for clients, we defined effective as one if they thought online counselling was efficacious. We applied the same definition to the client’s perception of the effectiveness of online counselling. Quantitative variables were included as independent variables in the regression and odds ratios were recovered.

## Results

The findings for both quantitative and focus group discussions (FGD) conducted on the effectiveness of online counselling during a pandemic is presented under themes that were derived from the research objectives. The themes were sub-divided to furnish relevant data, as contained in the questionnaires and FGD schedules.

### Demand for counselling

In order to establish the views and experiences of therapists on the online counselling service provided to clients, the researcher sought to investigate the demands for counselling from patients before and during COVID-19.

**Fig. 1:**
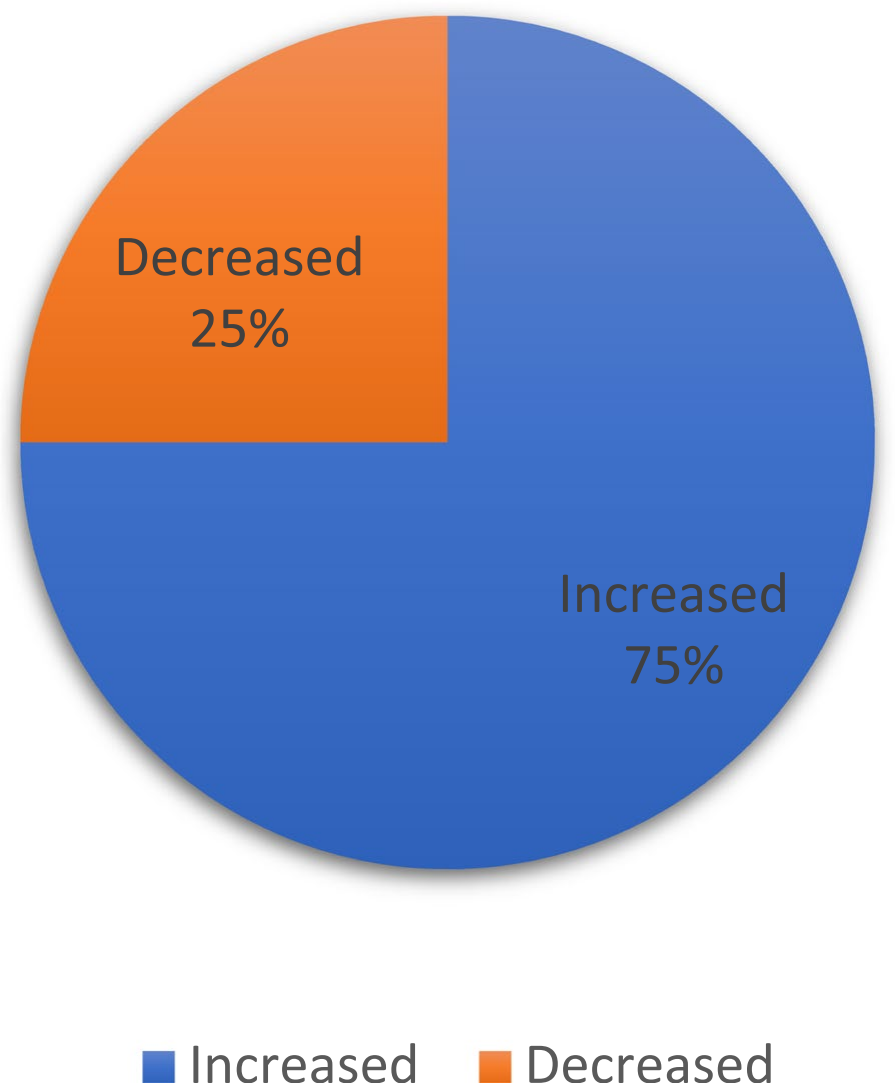
Change in the level of service demand before and during the COVID-19 Pandemic

Figure 1 shows that 75% [33] of the therapists reported that they experienced an increased demand for their services during the COVID-19 pandemic, whereas only 25% of the therapists experienced a reduced number of clients. Furthermore, most therapists who experienced an increase in demand indicated that the higher demand was especially from women, representing 54.5% of all clients.

### Level of preparedness to offer online counselling services

**Fig. 2:**
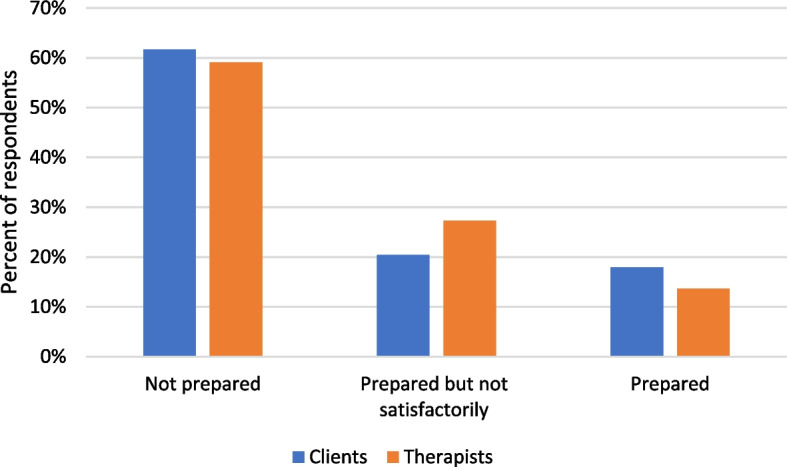
Clients and therapists’ evaluation of their preparedness to offer online counselling

Figure 2 shows that most (61%) clients indicated that therapists were not prepared to deliver counselling services online, while only 18% were of the opinion that their therapists were ready to offer counselling services online. The therapists also recorded similar percentages: about 58% said they were not prepared, while only about 14% said they were prepared. Despite these low levels of preparedness for online counselling, most therapists started offering services during the second wave of COVID-19.

During the focus group discussion, the researcher sought to understand why participants thought their therapists were not adequately prepared to offer online counselling. The client had several views, including confusion over the platforms to be used and a general lack of consistency. The participants had the following:

*"It's very easy to tell that one is not ready, you can see from the manner of delivery, the tools to be used; we kept changing the Apps to use for our interaction from video to calls to WhatsApp. It was not like it is for my online class at the university where you have a dedicated portal for the classes."* (Male client)

*"Check, yes we can understand when there is a dedicated platform but only challenges with Internet, but for my therapist, we had to do trial and error. We ended up just having short sessions on phone calls after trying a number of Apps."* (Female client)

However, the views that most therapists were not prepared were not universal. Some clients spoke highly of their therapists preparedness and generally ranked them as highly prepared. An example is a male client who was happy with their therapist as quoted in the text below:

*"Well my therapist knew what he was doing, and we only had a few disruptions in connection. We used Zoom, and our sessions were 30-40 minutes in the evenings. However, some other things, such as forms, were sent to me on WhatsApp for me to fill out and send back. In my view, he was generally prepared."* (Female client)

In contrast, therapists had different views regarding readiness. They stated that this was a new area and compelled them to undergo a learning process. Some had been used to on online platforms as attendees, but finding the best way to link with clients involved trial and error. This is captured in the statement:

*"Personally, during that time, I attended a lot of meetings online and they were no challenges so when I was receiving calls and requests from everywhere, I decided to jump on online counselling through Zoom. I did not put any special modalities prior to starting, but with time, I learned from the process. I found myself switching technologies just to try and meet up the shortfalls in one or the other technology adopted"* (Male Therapist)

Another therapist added.

*"In the context of your question, I did not prepare adequately for the service, I simply used the resources available and were in use at the time."* (Female Therapist)

### ICT applications explored for the provision of online counselling during the pandemic

The Table 1 shows that most of the 114 counseling sessions (40.1%) were conducted using video conferencing. The second most common tool was phone calls, whereas instant messaging applications were the least used. FGDs revealed that all respondents used multiple tools owing to the challenges in one or the other.

**Table 1:** ICT Media Used to Provide the Online Counselling

	Frequency	Percent
Chat Room (Instant Messaging)	21	7.4
Phone Calls	88	31.0
Video Conferencing	114	40.1
A Combination/ Alternating Tools	61	21.5
Total	284	100.0

During the focus group discussions, the following were the submissions by participants (clients) regarding the question of the technology used:

*"Well, I can't specifically say one technology was used, it was dependent on what worked on that day. What I mean is you would agree to use video conferencing but when you start the zoom call or google meet call, you find that you can't understand each other due to time lag or network connectivity so we were forced to switch to either call or just reschedule the session."* (Male Client)

*"For me it would start with WhatsApp where I receive the Link for a Google Meet call or Microsoft Teams, then we shift and have our discussion. Sometimes, we had to do a back and forth between video calls and text on WhatsApp, where we shared documents and assessment tools. But of course, my most preferred was a zoom call because I could see the person am talking to, and that just gave me assurance that what we are doing is serious. Unfortunately, my most preferred technology was the most challenging one to use and was very expensive."* (Female client)

We also asked the therapists what influenced the choice of technology to use. The study (see quotes below) highlights that the choice of technology platform for online counseling sessions during the pandemic was influenced by both pragmatic and preferential factors. For therapists, existing familiarity with a technology, based on prior use, was a key driver selecting media like WhatsApp that enabled multifaceted communication. This provided assurance for smoothly conducting sessions despite physical distancing requirements. Clients’ access and capabilities also dictated options, with video preferred but phone sessions necessary for that lacked internet connectivity. Despite differing circumstances across clients, both therapists and the majority of clients leaned toward video platforms as the optimal medium due to perceived benefits like enhanced engagement. However, network reliability and individual preferences occasionally necessitated relying on alternative channels. The findings reveal adaptability and client-centered considerations against the backdrop of pandemic response, though video counseling was regarded as most impactful by participants on both sides.

*"The technology was dictated by the type of client that I have on that day. If I have someone and they can only do phone calls, then I am forced to use the phone for the session; for those who would have access to the Internet, I would have options and enjoy the luxury to choose which one will best meet the objective of the service being sought. However, there are a few occasions when the choice is influenced by other factors such as network or client preference. The majority of my clients, including myself, seemed to prefer video conferences. It had challenges but it was still the best."* (Female Clients)

*"For me it was what I had used before because it gave me an idea how to go about the session. I did not want to have challenges that I did not know how to go about solving in the presence of my clients. My personal preference was WhatsApp because you could do many things on WhatsApp. Talk of video call, online voice call, share documents or give instant messages which actually show the client has read or seen my submission."* (Female Therapist)

We also asked the therapists about their experience with video-conferencing technology and stated that clients really preferred this method. One therapist stated:

*"Video conferencing was good especially for clients that thought we were just scammers and not properly trained and licensed. We could use it to verify most of these and to see the client to gain more insight about what is at hand. The biggest challenge which I believe was two sided was more bundles required and pathetic connectivity."* (Female Therapist)

### Limitations faced by clients and therapists using online counselling

#### Challenges faced by therapists

**Fig. 3:**
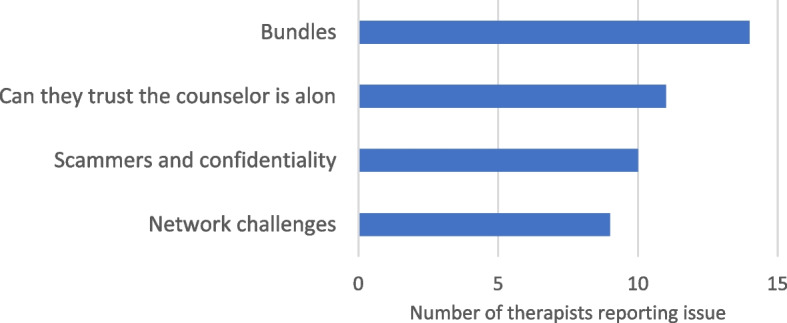
Challenges faced by therapists in providing online counselling

Therapists enumerated numerous challenges, but going by frequency count (Fig. 3), the following emerged as factors that decreased the effectiveness of online counselling service delivery. The most reported issue was the challenge of buying data bundles (bundles) to access the internet. Unlike in developed countries, where there is almost universal access to WiFi, accessing the Internet in Africa is costly [34]. This is consistent with what Gaved et al., (2020) stated regarding Zambia: internet access is too expensive or unreliable [35]. The second issue related to clients’ lack of trust and confidentiality was maintained during the sessions, with most clients questioning whether the counsellor was alone. Issues of being suspected of being scammers were also reported as well as the challenges of poor connectivity. Other issues that emerged during the discussion included complex problems owing to challenges with assessment and post-counselling monitoring because there were no physical visits, clients missing scheduled counselling sessions, difficulties scheduling, and forecasting the direction of the therapy progress because they could not fully assess the clients online.

For the question of network connectivity, we also asked which network was the most challenging with connectivity. One of the therapists advanced the following:

*"It was difficult to tell whose network was a challenge since we both needed connectivity. I personally do not remember asking my client what network they are using. So, it's safe to say, generally all networks had issues because connectivity was a problem for most clients I had."* (Male Therapist)

We asked the therapists about their experience with instant messaging and found that not many participants preferred texting. How often did they receive a request for counselling via a text message on their Facebook or WhatsApp accounts?

*"Mostly, I would receive my request for counselling or therapy via instant messaging either on my FB page, my WhatsApp number or a phone call. Sometimes through that same call, we would have a long talk and next we start setting up sessions with hopes of using different technologies such as Google Meet or Zoom... Texting and phone calls were manageable by almost everyone without much challenges but most people just wanted to see the 'therapists' thus very few often showed interest in phone counselling. But we used it as a backup when we experience challenges."* (Male Therapist)

Digital literacy was one of the other challenges that came out of the FGDs. We found that many experienced therapists were not computer literate, and this defeated the whole concept of introducing online counselling in such a difficult situation, which called for expertise and experience. One therapist stated:

*"Most of us counsellors are not computer literate therefore making it difficult for us to participate effectively in the digital world. As most of us are still comfortable with the traditional face-to-face kind of providing counselling, yet the world is moving from that aspect."* (Female Therapist)

#### Challenges faced by the clients

**Fig. 4:**
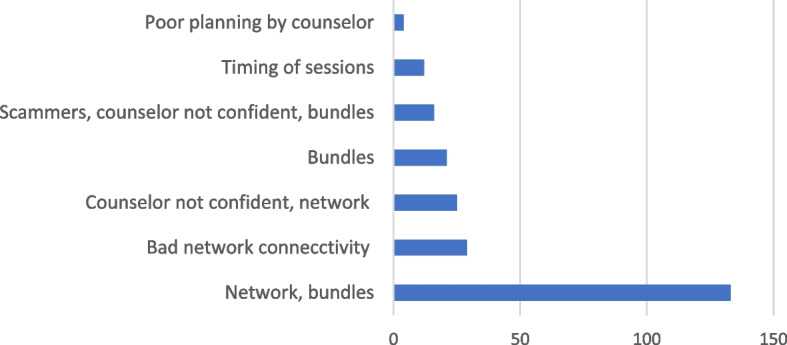
Challenges receiving counselling using online platform: views from clients

The clients listed a number of challenges that were also subjected to frequency counts to retain only a few strongly held perceptions. Figure 4 summarizes the findings gathered from the questionnaire on the challenges faced by clients receiving online counselling services. According to the questionnaire results, the most common issue was the network and limited bundles for connecting to online platforms. Other issues included privacy and lack of confidence by the therapist while using the platform.

The following were the main challenges expressed by clients during the FGDs: the multiplicity of media/applications used in service delivery resulting from poor network connectivity; insufficient funds for bundles, especially for the most perceived efficacious media, video conferencing, and not having an established platform for the services as it seemed a trial and error venture; Confidentiality of information they share with a total stranger they only meet virtually.

On the question of individual experience in video conferencing technology, clients stated that it had potential and was the best option; however, it was also riddled with many challenges, including poor network and costs. The clients had the following characteristics:

*"It is the most promising technology for me as it gives assurance, I was able to see who is with the therapist in the room and is the closest to the normal therapy sessions. However, it required more bundles, network was a challenge and was limited to 40 minutes since we were using free version for Zoom."* (Female client)

*"It was very irritating when the call cuts and a new link has to be created for us to continue the session. This was despite the breakages experienced. The whole thing was just making me anxious."* (Female client)

Regarding the issue of poor connectivity, the researcher sought to establish that there was a particular network that was the most challenging with connectivity. One client stated that:

*"The network was generally bad because I tried out using bundles for my two SIM-card for popular network providers but there was no much change. Am not sure but it's even possible network was bad on my therapist side"* (Male client)

The researcher also probed the focus group discussion participants about their experience with instant messaging, and the outcomes showed that not many clients preferred texts, but not many who used the option of texts. However, those who used it found the text to be confidential and fast. These are some of the responses.

*"I didn't use much of text messages except when setting up sessions or when receiving documents to work with"* (Male client)

Another man said, ‘Texting especially via WhatsApp was not the main medium of giving the therapy sessions but was just a backup and so I had no challenges with it.’ (Male client).

*"I preferred WhatsApp instant messaging because it was fast and also the person talking to me was unknown to me and didn't know me so I could open up.”* (Female client)

### The effectiveness of online counselling

**Fig. 5:**
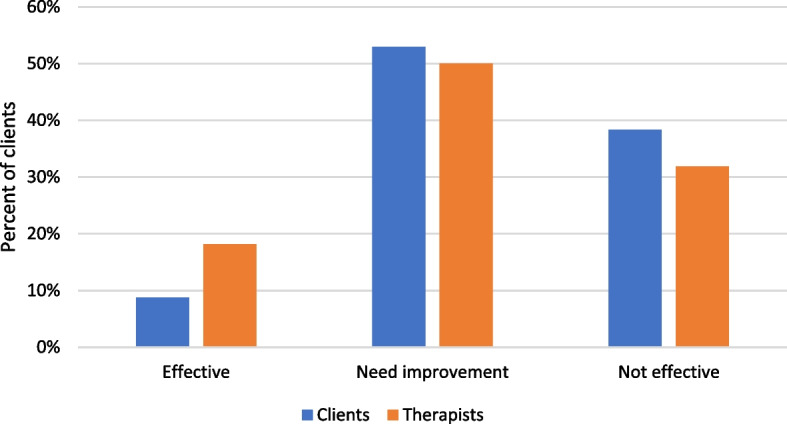
Clients’ and therapists’ perceptions of the effectiveness of online counselling

Figure 5 shows that a majority (53%) of the clients were not satisfied with online counselling as they called for improvement, with about 30% saying it was not efficacious in addressing their problems. Only a small proportion (9%) of clients thought it was efficacious. Fifty percent of the therapists indicated that online counselling needs improvement, 32% saw it as not efficacious, and the rest thought it was efficacious. The proportion of therapists who saw online counselling as efficacious was slightly higher than that of clients. Overall, the majority of clients and therapists did not see online counselling as efficacious.

**Fig. 6:**
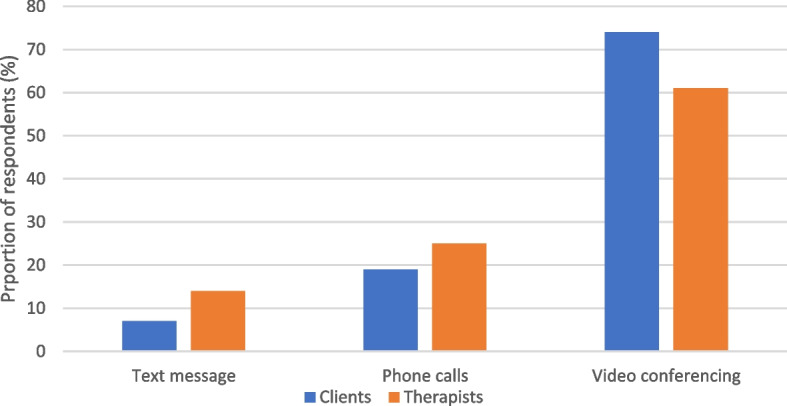
Clients and therapists views on tools considered efficacious

Narrowing down to the tool considered most efficacious (Fig. 6) in delivering online counselling, there was agreement between clients and counsellors that video conferencing tools were the best followed by phone calls and that the least efficacious were text messages. This presented a challenge for both clients and therapists, given that video conferencing used more bandwidth and affordability.

### Factors associated with a positive perception of online counselling

We began by exploring the factors associated with therapists’ preparedness and a positive perception of online counselling efficacy. Table 2 presents the estimated logistic regression results. According to the logistic regression analysis, we identified several factors associated with (a) preparedness of therapists to use online counselling platforms and (b) the effectiveness of online counselling services. There was a significant association between positive perception and belonging to the age group 26–37 years (OR 2.9, *p* < 0.05) compared to the reference group of 18–26 years. However, the results showed that older therapists (57–64 years) had significantly lower odds of being prepared for online sessions. Being in Lusaka (the capital) was associated with higher odds of categorizing online counselling as efficacious. This could be because of better connectivity in Lusaka than in other small towns. There was also a strong association between preparedness and profession. Specifically, the analysis showed that mental health nurses and psychiatrists had high odds ratios, but these were also not statistically significant.

**Table 2:** Factors associated with therapists’ preparedness and positive perception of online counselling: logistic regression

	(1)	(2)
VARIABLES	Prepared (0/1)	Efficacy (0/1)
Sex (1 = Male)	-3.718** (1.593)	1.012 (1.101)
Age (reference: 18–26 year)		
27–36 years	1.999 (1.273)	0.907 (1.296)
37–46 years	2.003 (1.702)	2.903** (1.481)
57–64 years	-2.834* (1.621)	2.664 (1.819)
Private practice (Reference: public)	-1.512 (1.198)	0.799 (1.282)
Lusaka (reference: other towns)	0.597 (0.977)	2.143** (0.978)
Profession (reference: Religious leaders)		
Lay counsellor (0/1)	2.034 (1.994)	-3.507** (1.373)
Mental Health Nurse	2.356 (5.054)	
Psychiatrist	5.570** (2.576)	-0.006 (1.619)
Psychologist	-1.607 (1.417)	-0.520 (2.697)
Psychosocial counsellor	-2.559* (1.346)	-1.322 (1.104)
Social worker	-0.382 (1.502)	
Constant	0.519 (1.468)	-0.671 (1.299)
Observations	44	37

Robust standard errors in parentheses

^***^p < 0.01

^**^p < 0.05*

^*^p < 0.1

On the client’s analysis, we tested for the association of demographics, media used, and experience with counselling with the effectiveness of online counselling services (Table 3). The results show that older clients (age group 45–50) have lower odds of perceiving online counselling as efficacious. Females had higher odds of having a positive perception compared to male clients. Phone calls were about three times more likely to be perceived as efficacious than other ICT tools that were used. However, the most important factor determining the effectiveness of online counselling from the clients’ perspective seems to be therapists’ preparedness for the session. The odds of perceiving online counselling as efficacious barely changed when the counsellor was not prepared and when prepared, but not satisfactorily. However, when the counsellor was prepared, online counselling was three times more likely to be perceived as efficacious compared to when the counsellor was not prepared.

**Table 3:** Factors associated with client’s positive perception of online counselling: logistic regression

	(1)
VARIABLES	efficacious
Age of client (Reference: < 25)	
25–30	0.592 (0.656)
31–34	-0.287 (0.914)
35–40	0.143 (0.674)
41–44	0.002 (0.724)
45–50	-1.586* (0.932)
> 50	-1.423 (0.867)
Sex (1 = Female)	0.842* (0.445)
Taken counselling before COVID	0.486 (0.450)
ICT tool used (Reference: other)	
Phone calls	2.802* (1.607)
Texting (0/1)	-1.850 (1.537)
Video conferencing (0/1)	0.638 (1.591)
Counsellor prepared but not satisfactorily (0/1)	-1.058** (0.494)
Therapist prepared (0/1)	2.997*** (0.979)
Constant	0.325 (1.332)
Observations	240

Robust standard errors in parentheses

^***^p < 0.01

^**^p < 0.05

^*^p < 0.1

### Views on online counselling during a pandemic

To further understand the challenges and success of online counselling, the researcher sought to obtain participants' overall opinions, especially during an outbreak. The shift to remote counseling during the pandemic was viewed as a necessary yet imperfect adaptation. Clients experienced heightened stress and anxiety in already difficult times when attempting to access care online, citing a lack of systemic investment and support as barriers. Therapists recognized online counseling's imperative role for continuity of mental healthcare provision amidst physical distancing policies. However, they noted high drop-out rates and inconsistent attendance compared to in-person sessions, indicating suboptimal effectiveness of current virtual practices [36]. Both clients and providers highlighted the need for dedicated applications, regulations around requisite qualifications and facilities, and ongoing optimizations to stabilize remote therapy delivery. Though the emergency transition was seen as having some merits, consensus indicated that further enhancements to capacity, quality assurance, and user-centered design would be essential for accessible, ethical, and impactful online mental health services going forward. We summarize the thoughts of both clients and therapists that support this conclusion.

Clients had the following opinions:

*"Normally, we would see a counsellor either at church or school or even just a doctor at hospital. But the Covid-19 pandemic imposed restrictions that brought more fear to us; the disease was new then we heard and saw people dying some of our loved ones, then we began to experience some of the same signs and symptoms. We needed this service, but it could not be accessed physically, so we decided to reach out online. Unfortunately, not much was done to support this avenue by the therapist or even the government. I can generally say this is viable but from my experience, it was not effective as it brought stress anxiety in trying to get help instead of helping."* (Female Client)

*"Let them make a special Application like what those in education have for e-learning. This try and error was not serving the purpose."* (Male Client)

The therapist had the following opinions.

*"This area needs investment to reach a desired standard. You can imagine the outbreaks we have as a country: Lusaka, for example, is known for cholera, the family involved requires support, but this may not be provided physically. Therefore, it becomes imperative for online services are essential. If possible, there should be regulations on who can offer online services depending on the equipment and facilities they have. In its current state, I feel that we are not justice to the system. Looking at the dropouts and the inconsistency in attending sessions, I can safely say we are not yet there."* (Female Therapist)

*"For me this was a learning experience, when I just started I had challenges and yes if I was to judge at that time I was going to say this was not effective. However, after seeing all the adjustments I have made to improve my services, I can confidently say it is effective. We rushed into it, but with time, we started to stabilize. I also agree with the previous speaker that submitted the need for regulating who provides these services not only based on facilities but qualification and experience too."* (Male Therapist)

## Discussion and implications

The purpose of this research was to examine and validate the opinions of clients and therapists regarding the effectiveness of online counseling in times of pandemics in Zambia. This study focused on their level of readiness, the method of providing counseling services, and the challenges they encountered. We found that most therapists were not ready to adopt online services when the COVID 19 pandemic hit. The common methods of connecting included video calling and voice calling. Both therapists and clients reported several challenges connecting online, therapist also faced trust issues and limitations relating to organizing and maintaining therapy sessions online. Worries regarding the ability to establish a connection with patients through online platforms seemed to have the greatest influence. These concerns were found to be a significant predictor of negative attitudes toward online therapy and how effective it was perceived to be.

### Level of readiness for online counselling

The study found that therapists were not prepared to offer online counselling services, but were compelled by high demands for counselling services from clients during the pandemic. Therapists were ill-equipped to offer online counseling services during the COVID-19 pandemic, even though they began providing such services in the second wave of the outbreak. This research discovered that the effectiveness of a therapy session, as perceived by the client, was influenced by how well-prepared the therapist is. It was discovered that therapists who were older had a lower level of readiness, and clients who were older had a diminished perception of the effectiveness of online counselling. While younger generations may have more familiarity with video conferencing, mostly for personal communication, older therapists with more clinical experience may have an advantage in developing more reliable and adaptable therapy skills that can be applied to the new online platform.

### Method of providing counseling services

This research discovered that different Information Technology tools were utilized, such as video conferencing, text messaging, phone calls, and a mix of these technologies, in response to the difficulties encountered. Earlier research has indicated that online counseling, specifically videoconference counseling, can be comparable to in-person counseling. This is because it helps enhance the motivation of individuals seeking counseling by allowing them to communicate with a psychological counselor in real-time [37]. Furthermore, it is possible to form therapeutic connections through online platforms that allow video and audio connection [29, 38]. Previous research conducted in developed nations has indicated a rise in the utilization of video conferences, computers, and telephones/smartphones. The main way in which computers and smartphones were utilized in our study aligns with what previous research has indicated. Our study's psychologists primarily employed video conferences and telephone calls, however due to connectivity problems the therapist had to resort to text and emails to conclude sessions and do follow up. A previous study found that email was the most frequently utilized method for online therapy [39].

### Challenges encountered during online counselling

The research discovered that therapists encountered various difficulties due to problems regarding evaluating and monitoring progress after counseling sessions. Other concerns included the environment or safety, lack of trust from some individuals who viewed them as frauds, issues with network connection and accessibility, clients not showing up for scheduled counseling appointments, and challenges in planning and predicting the course of therapy progress. Previous research discovered that online counselling also presented certain drawbacks, such as constraints related to technology [27–29, 40]. In the same way, another research paper listed the factors contributing to dropouts which included being overwhelmed by work responsibilities or having an excessive amount of work hours, lack of motivation, and skepticism about the effectiveness of the techniques employed in the group [41]. In our findings we also record the issue of distractions during sessions these included the reduction in excitement, or not focusing on the therapy session. One such common occurrence is known as zoom fatigue [42]. Clients complained about the use of multiple media and applications for service delivery, which was a result of poor network connection and limited funds for data packages. This was particularly problematic for the most effective media, such as video conferencing. Other issues from the clients included worries about privacy and confidentiality.

### Effectiveness of online counselling

This study found that both clients and therapists believed that online counselling was ineffective during the pandemic and needed to be enhanced. According to our findings, the online service did not impress a majority of the respondents, with a percentage of 89% citing ineffectiveness. This is contrary to what other studies found, for example, [43] discovered that the majority of psychotherapists expressed a moderately favorable opinion towards online psychotherapy, indicating that they were inclined to utilize online psychotherapy in the coming times. There is increasing evidence indicating that both online and in-person therapies have similar effectiveness in treatment [44, 45]. When the pandemic started, therapists were unfamiliar with conducting therapy online and did not have the necessary training or understanding of how effective it could be. This might have resulted in a more unfavorable first impression. Over time, as therapists have gained more experience in conducting online therapy, their perspectives have become increasingly optimistic [43].

### Limitations of the study

The limitation of our study is that we did not consider defining the indicators for effectiveness to our participants, hence there is a possibility that they may have had different interpretations of what effectiveness meant for them. We assumed that since respondents were selecting answers from given options, there would be no bias on how effectiveness is defined. We tried to give them response options which guided them towards the specific measures that we applied to define effectiveness (such as ICT tools, internet access, etc.). There was however one question (asking how efficacious online counselling was), this particular question may have led the respondents to derive their own indicator of effectiveness of online counselling. This could have been avoided by setting out clear indicators of effectiveness before drawing up the questionnaire, and then building our questions around these indicators. We may have attempted this but not 100%. Hence we recommend future studies to consider investigating the indicators of effectiveness or effectiveness.

## Conclusions

Online counselling had the potential to help alleviate the challenges caused by the COVID-19 pandemic among mental health patients who needed to access a therapist during restrictions. Despite its benefits, online counselling in Zambia came at a time when most therapists from Zambia where not prepared to handle the technicalities of online platforms. Hence our major finding was that participants found online counselling to be inefficacious. Key constraints came from the fact that most therapists where not tech savvy, particularly the old aged therapist. However, young therapist and those operating from Lusaka had a high level of proficiency in using the online services. For the clients, this was an appreciated solution but they had to deal with hurdles in the form of network connectivity and high costs of data. Video conferencing and voice calls were the most common methods used. These tools offered the best format for online counselling to the therapists and clients. These findings offer insight into the challenges that a developing country can face in trying to implement solutions that work in the developed countries. A key concern is that our infrastructure is limited and our preparedness to adopt these solutions is hindered by our lack of capacity development. There is need for training institutions to capacitate trainee/qualified therapists with digital skills required for online counselling. We also recommend developers, health researchers, government and the private sector to work on developing dedicated online counselling platforms which are affordable and offer privacy for the clients.

### Supplementary Information

**Supplementary Material 1.**

**Supplementary Material 2.**

## Data Availability

The data reported and supporting this paper were sourced from existing literature and are therefore available through a detailed reference list.
