# Supplementary material for: Effectiveness of online counselling during COVID-19 in Zambia: clients' and therapists' perspectives
Source: BMC Psychol. 2024 Mar 8;12:132. doi: 10.1186/s40359-024-01614-y (PMC10921579; doi:10.1186/s40359-024-01614-y)
Supplement: Supplementary file 1 — Supplementary Material 1. [file 40359_2024_1614_MOESM1_ESM.docx]

**Supplementary 1. Questionnaire**

RESEARCHER: **CHOONGO MULUNGU**

TITLE OF PROJECT: **Effectiveness of Online Counselling During COVID-19 in Zambia: A Clients and Therapists Perspective.**

Hello, my name is Choongo Mulungu, I am a Researcher and Mental Health Officer under the Ministry of Health based at Lusaka District Health Office. I am contacting the named study for the purpose of improving service delivery, informing policy formulation and providing a basis for future research. I would like to take a few minutes of your time to participate in this study whose long-term benefits would reflect back on you in one way or another as highlighted in the consent form. We use the term "online counseling services" to mean any type of counseling or mental health as well as substance abuse or coaching using telecommunication technologies such as internet, telephone, video conferencing or email. It is useful if you answer every item; however, you can skip any item except those marked with an asterisk (*). There is no right or wrong answer to the survey.

***(Please note that the researcher will put a determinant for proceeding or discontinuing with the study by use of these questions. This will be so to fulfil the exclusion criteria in which only those clients that would have attended counseling services online will be allowed to proceed and only therapists that provided online counseling services during the period under review would proceed with the questionnaire)***

1. I am 18 years of age or older (this is a required item) Yes or No
2. I am a mental health professional currently providing counseling services (this is a required item) Yes or No
3. I have read and understood the informed consent form and agree to participate in this study (this is a required item) Yes or No
4. What is your age? *

18-26

27-36

37-46

47-56

57-64

65+

1. Select the district you are working from*

Drop down (with districts in Zambia for one to select)

1. What is your gender *

Male,

Female,

Other

1. What is your primary work setting? *

Independent practice

Small group practice (2-10 practitioners)

Large group practice (10+ practitioners)

Health or mental health facility

Hospital

Military

University

Other

1. What telecommunication tools have you used to deliver counseling during the epidemic? (click all that apply)

Mobile phone

Chat room or instant messaging

Email

Video conferencing (zoom, google meet, teams)

Smart phone application e.g. WhatsApp

Other Specify

1. What age group did you serve predominantly?

Children

Adolescents

Adults

Elderly

1. What is your profession?

Nurse

Marriage and family therapist

Social worker

Psychologist

Psychiatrist

Psychosocial counselor

Physician

Other

1. Do you think it is ethical for a licensed mental professional to deliver counseling services online?

Yes

No

Unsure

1. Are you aware of any laws or regulations that govern delivery of counseling services?

Yes

No

1. Which of the following communication technologies do you consider useful for online counseling?

Email

Texting

Telephone (mobile or landline)

Video conferencing

Other Specify _________________________________________

1. Do you think mental health practitioners should undergo any training about technical issues of telehealth?

Yes

No

Unsure

1. How comfortable were you with delivering online counseling services?

Not comfortable

Slightly comfortable

Moderately Comfortable

Very comfortable

1. How confident were you in providing online counseling services without initial in-person assessment?

Not confident

Slightly confident

Moderately confident

Very Confident

1. How many hours per week on average were you delivering on-line counseling services?

1-5

6-10

11-15

16-20

21-24

24+

1. Did you have more clients or less during the epidemic?

More

Less

Not sure

1. By what percentage was your increase or reduction in clients?

Specify _______________________________________________

1. What concerns do you have with online counseling during epidemics? (select what applies)

Security/ confidentiality

Lack of adequate preparedness/ inability to handle emergency situations

Equipment

Clients missing sessions

Lack of training

Licensure issues

Lack of support in research

Don’t have any concerns

1. What concerns did your clients have over online counseling services?

List ____________________________________________________

1. How did you manage their concerns over the online counseling?

Specify ______________________________________________________

1. Do you think online counseling is efficacious in our country?

Yes

No

No sure

1. What do you suggest should be the method of providing counseling services during epidemics?

Online

In person

***The End.***

Note: this is just a format of questions but the online too will have two different links one for therapists and the other for clients.
