# Supplementary material for: Effectiveness of online counselling during COVID-19 in Zambia: clients' and therapists' perspectives
Source: BMC Psychol. 2024 Mar 8;12:132. doi: 10.1186/s40359-024-01614-y (PMC10921579; doi:10.1186/s40359-024-01614-y)
Supplement: Supplementary file 2 — Supplementary Material 2. [file 40359_2024_1614_MOESM2_ESM.docx]

**Supplementary 2. Focus Group Discussion Guide**

RESEARCHER: **CHOONGO MULUNGU**

TITLE OF PROJECT: **Effectiveness of Online Counselling During COVID-19 in Zambia: A Clients and Therapists Perspective**

Hello, my name is Choongo Mulungu, I am a Researcher and Mental Health Officer under the Ministry of Health based at Lusaka District Health Office. Welcome to our discussion whose purpose is improving service delivery, informing policy formulation as well as providing a basis for future research on the same subject.

Let me just remind you that participation in this discussion is entirely voluntary and will attract no compensation. Further, I would like to request that we respect each other’s views and opinions. Should you see the need to react to another person’s submission, you will be allowed to do so without demeaning the one that advanced the submission. It is the objective of this discussion to gain consensus and agreed positions about the themes and this will be achieved through controlled debates. It will also be acceptable if there will be any issue which may not gain a general conclusion in terms of consensus. Take note that names are not necessary in this discussion.

We will use the term "online counseling services" to mean any type of counseling or mental health as well as substance abuse or coaching using telecommunication technologies such as internet, telephone, video conferencing or email.

The following are the guiding primary questions

1. What prompted you to use the online counseling services?
2. Did you have any options available to get the help you wanted?
3. Is it common that people use this mode to access services?
4. How did end up with the counselor or therapist that attended to you
5. What Information Technology (IT) tools did you use for the therapy sessions?
6. What was your experience before, during and after the sessions?
7. From your experience, what was the most effective IT tool
8. What Challenges did you face to get the help you needed through counseling?
9. I understand you were attended by different therapists, in relation to the one that attended to you, how was their level of preparedness?
10. Who decided upon the tools to be used for the sessions?
11. How was their mastery of IT tools they used?
